# Diagnosing Different Types of Bacteria on Culex Mosquito Spp. (Diptera: Culicidae) in Baghdad

**DOI:** 10.7759/cureus.33177

**Published:** 2022-12-31

**Authors:** Raghad Khalaf Ibraheem Aljoboory, Saeed Maher Lafta

**Affiliations:** 1 Department of Biology, College of Education, AL-Iraqia University, Baghdad, IRQ; 2 Department of Biology, College of Science, Tikrit University, Tikrit, IRQ

**Keywords:** culicidae, diptera, bacteria, bacillus, culex

## Abstract

Introduction

The genus of mosquitoes is also one of the important factors affecting the diversity of bacteria; the aim of the current research is to isolate the types of bacteria from adult mosquitoes that grow near stagnant water ponds in Baghdad Governorate, in order to collect information about bacterial species common in mosquitoes present in the area.

Methods

A number of Culicidae mosquito specimens were collected from Abu Ghraib, Baghdad, and the isolation of microorganisms from mosquitoes after prepared culture media and then a biochemical study of the identification characteristics of bacterial isolates was done.

Result

Some morphological traits were studied, and their production of catalase and oxidase enzymes was determined; 2×10^3^ bacterial cells/ml of different genera and species were isolated on a solid agar nutrient medium for insect models. Bacterial colonies appeared with shapes ranging from pinworms to colonies with flat edges, white color, and smooth surfaces, and some types of fungi were isolated with a number of 1×10^2^ cells/ml. Bacterial cells were isolated on the optional MacConkey medium, only less than 100 cells/ml when using the direct method, and the number reached 1×10^2^ bacterial cells/ml for some models. Many *Bacillus* isolates were isolated, and the cells that formed the spores appeared in rods of different sizes, and the shape and location of the spores differed from the mother cells, where the isolate *Bacillus* species appeared in small-sized cells with a medial cylindrical spore and in the form of thin rod-shaped cells with a cylindrical spore. The spores are large, medium in size, and from the mother cell, while the sporophyte took a sub-terminal position in isolate *Bacillus* spp. The sporophyte appeared in a barrel shape, the average of the parent cell in the isolate, and the spheroid was mediated by the parent cell, and the cells of the isolate were characterized by their small size and the location of the sporophyte under the two ends where the most isolated were *Bacillus*, Enterobacteriaceae species.

Conclusion

Several types of bacteria accompanying mosquitoes were isolated, *Bacillus* spp., *Staphylococcus*, and *Streptococcus faecalis*, which cause many diseases in humans and need molecular diagnosis to confirm the isolated species more accurately.

## Introduction

Mosquitoes belong to the family Culicidae and contain more than 3,500 different species worldwide [1]. Most of the studied and personal species belong to the genera *Aedes*, *Anopheles*, and *Culex* and are important in the food chain, as they are food for many birds and have a role in pollinating many plants in addition to their great impact on cleaning the environment. Despite all the advantages, mosquitoes have many blood-sucking feeding organs and are able to transmit pathogens to humans and animals, which is great in terms of public health [2].

Mosquitoes are among the most important arthropods that transmit diseases, the most important of which is the malaria parasite [3,4]. *Aedes* sp. and *Culex* sp. transmit diseases such as Japanese encephalitis (JE) and filariasis. Japanese encephalitis (JE), popularly called "brain fever," is caused by a virus. JE is a zoonotic viral disease. Filariasis, commonly called "hidden disease," is a disabling, disfiguring disease caused by nematode parasitic worms [4,5], especially through international travel and trade that have led to expanding the spread of mosquito species that were previously confined to certain areas. Studies have shown that the microbial communities isolated from the midgut of laboratory-reared fourth-instar *Culex tarsalis* (a vector of western encephalitis and West Nile viruses) using conventional culturing techniques included several species, such as *Lactobacillus*, *Micrococcus* sp., *Micrococcus candidus*, *Saccharomyces*, *Proteus rettgeri*, *Geotrichum*, *Pseudomonas*, and other unidentified gram-negative bacteria [6,7]. Most *Anopheles* species live in clear water exposed to sunlight, while *Culex* and *Aedes* are mostly found in stagnant or turbulent waters that contain a lot of organic matter [8].

The genus of mosquitoes is also one of the important factors affecting the diversity of bacteria, as male and female mosquitoes show different environmental behaviors in terms of feeding and dispersal capabilities. Both sexes feed on nectar and plants capable of decomposing sucrose, but females are also bloodsuckers to complete their reproductive life cycles. *Bacillus* and *Staphylococcus* were detected in males, while bacteria from the genera *Chryseobacterium*, *Pseudomonas*, and *Serratia* were detected exclusively in females [9,10].

The interactions between mosquitoes and their associated microbiota have yet to be investigated in depth. Most of the published studies describe bacterial diversity and how it varies according to particular factors. Nevertheless, a common conclusion is that a more comprehensive analysis of symbiotic mosquito interactions is needed at evolutionary and functional levels. Better knowledge of the biological impacts will enable the development of efficient biocontrol approaches for mosquito-borne diseases (MBD) [11].

## Materials and methods

Collect samples of the following insects

A number of live mosquitoes (Culicidae: *Culex* sp.) were caught from water ponds in the Abu Ghraib area on the outskirts of Baghdad Governorate during the month of March. The samples were placed in small, opaque plastic containers and transferred to the laboratory for the purpose of isolating microorganisms from them with an ethical clearance number IEC/2022/121 from Al-Iraqia University.

Isolation of microorganisms from mosquitoes

A number of mosquitoes were taken and weighed at 0.1 g; the outer surface of the mosquito was sterilized with 75% alcohol, which was left to dry and placed in a sterilized ceramic jar, and 10 ml of 0.85% physiological salt solution (NaCl) was added to it. The mosquitoes were mashed quietly, and the resulting solution was collected in a sterile tube for the purpose of complete isolation. A number of culture media were prepared according to the recommendations of the processing company: nutritious agar medium, solid MacConkey medium, and Vaser solid medium. The media were sterilized with an autoclave for a quarter of an hour at a temperature of 121°C and a pressure of 1.5 atmospheres, and the media were cooled to 50°C after the end of sterilization, then poured into sterilized plastic dishes that were left to solidify, and left for 18 hours to test for sterility; 0.1 ml of mashed insect solution was added to the surface of the dishes and distributed by a sterile glass diffuser. The dishes were left for 10 minutes in the laboratory atmosphere, then transferred to the incubator at 37°C, and incubated for 24-72 hours. Single colonies were picked from each medium and regrown individually by the method of planned multiplication on agar nutrient medium and incubated at 37°C for 24 hours. In order to isolate the spore-forming bacteria, a portion of the solution was taken from the mashed mosquitoes and placed in a water bath at 80°C for half an hour; then, 0.1 ml of the mashed insect solution was added to the surface of the dishes and distributed by a sterilized glass diffuser on the surface of the agar [12].

A second drop was also taken and mapped by the ABCD method on the surface of the nutrient agar and incubated at 37°C for 24 hours to obtain single bacterial colonies. The method of fortification was adopted to confirm the obtaining of bacteria by taking 1 ml of heat-treated insect pure, adding it to 10 ml of the nutrient broth medium, and incubating at 37°C for 24 hours, after which a drop was taken and plotted on the surface of the nutrient agar to obtain single colonies. The single colonies were picked and replanted on the nutrient agar medium for the purposes of propagation and character study [11,13].

Biochemical study of the identification characteristics of bacterial isolates

The phenotypic characteristics were studied on the used culture media, and the bacterial shape, texture, edges, and fermentation of lactose sugar were determined in the MacConkey medium. The phenotypic characteristics of the isolates were studied after staining them with gram stain to determine the microscopic shape and arrangement of cells and whether they are gram-positive or gram-negative. The presence and location of the sporophytes in the mother cell and the shape of the sporophytes of bacteria were determined in the study of its fermentation of lactose sugar on MacConkey medium, where the fermentation of lactose sugar leads to a change in the color of the medium from pink to yellow.

The study of the production of bacterial isolates of catalase and oxidase enzymes and their enzymes

The isolates under study are activated by culturing them on an agar nutrient medium and incubating them for 24 hours at 37°C. A little bacterial growth is taken with a wooden stick and placed on a clean slide, and a drop of hydrogen peroxide solution is added to it. The formation of air bubbles indicates the bacterial production of the enzyme catalase [14].

Bacterial growth is also taken with a wooden stick and spread on the surface of a small blotting paper. Drops of aqueous solution are added to the reagent N,N,N,N tetramethyl-para-phenylenediamine dihydrochloride, with a concentration of 1%, and the formation of a violet color indicates the production of isolates of the oxidase enzyme [15,16].

## Results

The primary isolation by the direct dilution method without the need for model enrichment showed the presence of 2×10^3^ bacterial cells/ml of different genera and species on a solid agar nutrient medium. Bacterial colonies appeared in both models with shapes ranging from pinworms to colonies with flat edges, a white color, and a smooth surface, and some types of fungi were isolated with a number of up to 1×10^2^ cells/ml. Bacterial cells were isolated on the facultative MacConkey medium when using the direct method for insect models but only less than 100 cells/ml and 1×10^2^ bacterial cells/ml for other models.

Heat treatment at 80°C of the insect mixture determined the presence of a number of spore-forming bacteria belonging to the genus *Bacillus*, and the number of these spore-forming species ranged from 1×10^2^ to 2×10^2^ cells/ml. Table 1 shows the results of the initial isolation of bacteria from adult mosquito (*Culex* spp.) samples.

**Table 1:** The tests for isolating bacterial species by the media used and the phenotypic characteristics of the isolated bacterial species

Formal characteristics	Insect model, 2 cells/ml	Formal characteristics	Insect model, 1 cell/ml	Test/medium used for insulation
(1) Colonies with flat edges and white color, (2) colonies with flat edges and transparent color, (3) pin colonies (predominant in the model), and (4) the appearance of fungi after continuing incubation for more than 72 hours	1.5×10^3^	(1) Colonies with flat edges and white color, (2) colonies with flat edges and transparent color, (3) pin colonies, and (4) the appearance of fungi after continuing incubation for more than 72 hours	2×10^3^; fungus 5×10^2^	Total number/medium of nutritious agar
The appearance of the bacteria in a pale pink color that does not ferment the sugar lactose	1×10^2^	Nothing	Less than 1×10^2^	Enterobacteriaceae isolation; Enterobacteriaceae in the middle of the MacConkey agar
-	No growth after 72 hours	-	No growth after 72 hours	The isolation of Enterobacter faecalis (incubation temperature of 44°C); Enterobacteriaceae in the middle of the liquid MacConkey agar
-	No growth after 72 hours	-	No growth after 72 hours	The isolation of Streptococcus faecalis/solid Vaser medium (selective medium)
Colonies of white color and smooth edges; colonies of wrinkled surface and uneven edges	2×10^2^	Colonies of white color and smooth edges	1×10^2^	The isolation of Bacillus/agar nutrient media and the heat treatment of the model

Biochemical test results

Some basic diagnostic characteristics were taken to determine the group of bacteria to which the isolates belong, including the examination of the production of the enzyme catalase, where the release of oxygen gas and the formation of air bubbles were observed when adding a drop of hydrogen peroxide solution to each *Staphylococcus*, which is taken as a factor of virulence for this bacterial type.

## Discussion

The microscopic examination revealed the isolation of *Staphylococcus* bacteria, which are spherical, staphylococcal, and positively gram-stained. The spherical, gram-negative, rod-shaped cells are representative of the *Enterococcus* group, and the cells forming the sporangia appeared in a different size, and the shape and location of the sporophyte differed from the mother cells, where the isolate *Bacillus* spp. appeared in small-sized cells with a medial cylindrical spore. Isolate *Bacillus* spp. appeared in the form of slender rod-shaped cells with a large, cylindrical sporophyte of medium size of the mother cell, while the sporophyte took a sub-terminal position in isolate *Bacillus* spp., and the sporophyte appeared in the form of a barrel shape and medium size of the parent cell in the isolate and the sporophyte mediation of parent cell. Isolation cells were characterized by their small size and the location of the sporozoites under the two ends [17,18].

The intestinal group gave a positive test for catalase. All isolates of *Bacillus-*forming spores were positive for the catalase assay, which is one of the most important characteristics that distinguish the genus *Bacillus* [8]. An examination of the production of the oxidase enzyme was conducted as a second confirmatory test for the groups, and *Staphylococcus aureus* appeared to be negative for the oxidase assay, as evidenced by the absence of a violet color around the bacterial culture after adding the reagent to it. The intestinal group was also diagnosed as negative for the oxidase production test, as it may be due to *Acinetobacter* bacteria other than *E. coli* because it does not ferment the sugar lactose in the medium of MacConkey. The confirmation of the diagnosis needs other tests that are outside the scope of this research.

While isolates of *Bacillus* spp. appeared to produce the oxidase enzyme in varying degrees, it is clear from the initial isolation that the bacterial pattern varies in adult *Culex*, and the reason for the lack of numbers or the lack of variation may be due to the stage of the insect or the environment in which it grows and the geographical location, type, and sex of the insect, and the method of isolation depends on the possibility of isolating different species where the direct method was adopted in isolation to determine the actual bacterial number and to avoid unmeasured numbers in the fortification method [9]; this explained the factors affecting the isolation of bacterial species from mosquitoes.

The study has limitations in that the bacteria tested are very selective and the sample was tested in vitro. Also, there is no test of antibiotic sensitivity, which can be taken into consideration in future studies.

## Conclusions

Understanding the mechanism(s) underlying why mosquitoes require live bacteria is important for managing animals that transmit diseases to humans since the processes involved may be susceptible to interruption. The study concluded that several types of bacteria accompanying mosquitoes were isolated, *Bacillus* spp., *Staphylococcus*, and *Streptococcus faecalis*, which cause many diseases in humans and need a molecular diagnosis such as DNA barcoding and xenomonitoring to confirm the isolated species more accurately.

## References

[1] Becker N, Petric D, Zgomba M, Boase C, Madon M, Dahl C, Kaiser A (2010). Mosquitoes and their control. Dordrecht, London, Moscow: Plenum Press.

[2] Cumberland SA, Lead JR (2009). Particle size distributions of silver nanoparticles at environmentally relevant conditions. J Chromatogr A.

[3] Galal FH, AbuElnasr A, Abdallah I, Zaki O, Seufi AM (2017). Culex (Culex) pipiens mosquitoes carry and harbor pathogenic fungi during their developmental stages. Erciyes Med J.

[4] Wang Y, Gilbreath TM 3rd, Kukutla P, Yan G, Xu J (2011). Dynamic gut microbiome across life history of the malaria mosquito Anopheles gambiae in Kenya. PLoS One.

[5] Gurunathan A, Senguttuvan J, Paulsamy S (2016). Evaluation of mosquito repellent activity of isolated oleic acid, eicosyl ester from Thalictrum javanicum. Indian J Pharm Sci.

[6] Becker N, Huber K, Pluskota B, Kaiser A (2011). Ochlerotatus japonicus japonicus-a newly established neozoan in Germany and a revised list of the German mosquito fauna. Eur Mosq Bull.

[7] Chao J, Wistreich G (1959). Microbial isolations from the mid-gut of Culex tarsalis Coquillett. J Insect Pathol.

[8] Lindh JM, Terenius O, Faye I (2005). 16S rRNA gene-based identification of midgut bacteria from field-caught Anopheles gambiae sensu lato and A. funestus mosquitoes reveals new species related to known insect symbionts. Appl Environ Microbiol.

[9] Kirschfink M, Mollnes TE (2003). Modern complement analysis. Clin Diagn Lab Immunol.

[10] Xue L, Manore CA, Thongsripong P, Hyman JM (2017). Two-sex mosquito model for the persistence of Wolbachia. J Biol Dyn.

[11] Bravo A, Sarabia S, Lopez L (1998). Characterization of cry genes in a Mexican Bacillus thuringiensis strain collection. Appl Environ Microbiol.

[12] Minard G, Mavingui P, Moro CV (2013). Diversity and function of bacterial microbiota in the mosquito holobiont. Parasit Vectors.

[13] Darboux I, Nielsen-Leroux C, Charles JF, Pauron D (2001). The receptor of Bacillus sphaericus binary toxin in Culex pipiens (Diptera: Culicidae) midgut: molecular cloning and expression. Insect Biochem Mol Biol.

[14] Clements AN (1999). The biology of mosquitoes. https://www.cabi.org/isc/abstract/19990505183.

[15] Pidiyar VJ, Jangid K, Patole MS, Shouche YS (2004). Studies on cultured and uncultured microbiota of wild Culex quinquefasciatus mosquito midgut based on 16s ribosomal RNA gene analysis. Am J Trop Med Hyg.

[16] Rani A, Sharma A, Rajagopal R, Adak T, Bhatnagar RK (2009). Bacterial diversity analysis of larvae and adult midgut microflora using culture-dependent and culture-independent methods in lab-reared and field-collected Anopheles stephensi-an Asian malarial vector. BMC Microbiol.

[17] Church DL (2016). Biochemical tests for the identification of aerobic bacteria. Clinical microbiology procedures handbook.

[18] Chandel K, Rasesh YP, Murlidhar JM, Yogesh SS, Vijay V (2015). Isolation and characterization of Vagococcus sp from midgut of Culex quinquefasciatus (Say) mosquito. J Vector Borne Dis.

